# Automated interpretation of cardiotocography using deep learning in a nationwide multicenter study

**DOI:** 10.1038/s41598-025-02849-4

**Published:** 2025-06-04

**Authors:** Chang Eun Park, Byungjin Choi, Rae Woong Park, Dong Wook Kwak, Hyun Sun Ko, Won Joon Seong, Hyun-Hwa Cha, Hyun Mi Kim, Jisun Lee, Hyun-Joo Seol, Seungyeon Pyeon, Soon-Cheol Hong, Yun Dan Kang, Kyung Joon Oh, Joong Shin Park, Young Nam Kim, Young Ah Kim, Yoon Ha Kim, Gwang Jun kim, Miran Kim, Hye Jin Chang

**Affiliations:** 1 Department of Convergence Healthcare Medicine, Ajou University Graduate School of Medicine, Suwon, Republic of Korea; 2 Department of Biomedical Informatics, Ajou University Graduate School of Medicine, Suwon, Republic of Korea; 3 Jeju National University Hospital, Jeju, Republic of Korea; 4 Department of Obstetrics and Gynecology, Ajou University School of Medicine, Suwon, Republic of Korea; 5 Department of Obstetrics and Gynecology, Seoul St. Mary’s Hospital, College of Medicine, The Catholic University of Korea, Seoul, Republic of Korea; 6 Department of Obstetrics and Gynecology, School of Medicine, Kyungpook National University, Daegu, Republic of Korea; 7 Department of Obstetrics and Gynecology, Kyung Hee University School of Medicine, Kyung Hee University Hospital at Gangdong, Seoul, Republic of Korea; 8 Department of Obstetrics and Gynecology, Korea University Medicine, Seoul, Republic of Korea; 9 Department of Obstetrics and Gynecology, Dankook University, School of Medicine, Cheonan, Republic of Korea; 10 Department of Obstetrics and Gynecology, Seoul National University Bundang Hospital, Seoul National University College of Medicine, Seongnam, Republic of Korea; 11 Department of Obstetrics and Gynecology, Seoul National University College of Medicine, Seoul, Republic of Korea; 12 Department of Obstetrics and Gynecology, Inje University Busan Paik Hospital, Inje University College of Medicine, Busan, Republic of Korea; 13 Department of Obstetrics and Gynecology, Ilsan Paik Hospital, Inje University College of Medicine, Goyang, Republic of Korea; 14 Department of Obstetrics and Gynecology, Chonnam National University Medical School, Gwangju, Republic of Korea; 15 Department of Obstetrics and Gynecology, Chung-Ang University Hospital, Seoul, Republic of Korea

**Keywords:** Cardiotocography, Deep learning model, Fetal monitoring, Medical research, Computational science

## Abstract

Timely detection of abnormal cardiotocography (CTG) during labor plays a crucial role in enhancing fetal prognosis. Recent research has explored the use of deep learning for CTG interpretation, most studies rely on small, localized datasets or focus on outcomes less relevant to clinical practice. To address these limitations, we developed a clinically applicable model using a large-scale, nationwide CTG dataset with reliable annotations provided by a board-certified obstetrician. Our study utilized 22,522 deliveries from 14 hospitals, each including cardiotocography (CTG) recordings of up to 75 min in length. The CTG signals were segmented into 5-minute intervals, resulting in a total of 519,800 person-minutes of analyzed data. We trained and validated a deep learning model based on CTG segments for classifying normal and abnormal CTGs. In the independent test dataset, the model achieved an AUC (area under the receiver operating characteristic curve) of 0.880 and PRC (area under the precision-recall curve) of 0.625 in internal tests. External tests across three datasets achieved AUCs of 0.862, 0.895, and 0.862 and PRCs of 0.553, 0.615, and 0.601. Our study results show the potential of the deep learning for automated CTG interpretation. We will evaluate this model in future prospective studies to assess the model’s clinical applicability.

**Supplementary Information:**

The online version contains supplementary material available at 10.1038/s41598-025-02849-4.

## Introduction

Cardiotocography (CTG) is an essential tool for real-time monitoring of fetal status during labor^1^, serving a critical function in detecting abnormal patterns by monitoring fetal heart rate (FHR) and uterine contractions (UC). Abnormal CTG waveforms may indicate fetal hypoxemia, and failure to detect fetal hypoxemia in a delays in timely detection elevate the risk of long-term complications for both the mother and fetus, making the accuracy and promptness of CTG interpretation clinically significant^2^. However, since most CTG interpretation is still performed manually by obstetricians, there are frequent delays in detecting abnormal patterns, which can adversely affect the safety of both the fetus and the mother.

Simple abnormal signals, such as bradycardia and tachycardia, are already alarmed by commercial devices that utilize traditional signal processing methods. However, traditional automatic CTG interpretation systems, which rely on basic machine learning or mathematical formulas, have shown limitations in addressing more complex and nuanced patterns, such as late variable decelerations. These limitations ultimately hinder their ability to fully enhance clinical decision-making and improve patient outcomes. For instance, a clinical trial conducted in 2017 confirmed that computer-based CTG interpretation did not enhance neonatal outcomes^3^.

Recently, with advancements in technologies such as deep learning, there have been numerous attempts to utilize models based on CTG raw waveform data. However, according to a scoping review published in 2024^4^, most prior studies have been conducted using data collected from a small number of institutions, resulting in insufficient dataset sizes. Except for studies conducted by Petrozziello et al. and McCoy et al., all studies utilized datasets with fewer than 5,000 patients^5–10^. Since many studies used data from only a few thousand patients, the performance of deep learning models has often been moderate, and insufficient external validation has limited the generalizability of these models.

Furthermore, most studies, including the one by Petrozziello et al., have focused solely on predicting fetal acidosis or similar outcomes. This approach is rarely used by obstetricians and, as a result, is difficult to apply in clinical practice. In typical clinical decision flows, CTG is utilized to assess fetal hypoxemia in real-time based on specific patterns. Identifying these patterns requires large-scale labeled datasets.

Our study aims to overcome these limitations by collecting nationwide, large-scale CTG data from multiple institutions. Additionally, a committee of obstetricians will collaborate to create a comprehensive labeled dataset, which will serve as the foundation for developing a high-performance deep learning-based CTG interpretation model. The model developed in our study is expected to be applicable as an automated CTG interpretation system in the future^11^.

## Materials and methods

### Data sources

Between January 2010 and December 2020, we collected a total of 22,651 delivery records from the delivery wards of 14 hospitals in South Korea.

The study cohort included patients who visited the hospital for delivery and underwent at least one fetal heart rate record in the hospital. Both singleton and multiple pregnancies were included. We collected cardiotocography (CTG) data along with relevant maternal demographics, such as maternal age and gestational age, as well as obstetric history, pregnancy complications, and neonate outcomes from electronic medical records (EMRs) of each hospital. We excluded cases if any of the following information was missing gestational age, Apgar score, mode of delivery (vaginal delivery or cesarean section), gestational weeks, or birth outcomes. We also exclude if only one fetal heart rate was available in cases of multiple pregnancy.

We utilized 17,494 singleton deliveries and 1,246 multiple deliveries from 11 hospitals for model development and internal validation. For external validation, data from three hospitals were included. External validation hospital 1 contained a total of 2,372 delivery records, among which 1,886 were singleton deliveries and 486 were multiple deliveries. External validation hospital 2 included 1,307 delivery records, comprising 1,135 singleton deliveries and 172 multiple deliveries. External validation hospital 3 consisted of 191 delivery records, of which 133 were singleton deliveries and 58 were multiple deliveries. This data collection project was funded by the National Information Society Agency (NIA).

The study was approved by Instituional Review Board(IRB) along with the Ajou University Medical Center, Seoul St. Mary’s Hospital, Kyungpook National University Chilgok Hospital, Kyungpook National University Hospital, Kyung Hee University Hospital, Korea University Medical Center, Dankook University Hospital, Inje University Paik Hospitals(Busan, Haeundae, Ilsan), Seoul National University Hospital, Seoul National University Bundang Hospital, Chonnam National University Hospital, Chung-Ang University Hospital. Institutional Review Board (IRB) approvals from all hospitals and further details can be found in Supplementary Note. Because our study was retrospective and personal information was anonymized, the institutional review boards of the aforementioned institutions waived the requirement for informed consent. Our study was subsequently designed and conducted in accordance with the World Medical Association Declaration of Helsinki and all relevant guidelines and regulations for medical research involving human subjects.

The CTG data were collected as PNG files, and information on fetal heart rate (FHR) and uterine contractions (UC) was extracted at a frequency of 0.5 Hz using the Hough transform algorithm^12^. Data with continuous interruptions in FHR or UC lasting more than one minute were excluded from the study. The total length of CTG data ranged from a minimum of 5 min to a maximum of 70 min. In this study, we minimized pre-processing to preserve clinically relevant information. Outliers and non-physiological signals were retained, acknowledging their potential significance. Only data segments with missing intervals exceeding one minute were excluded. We presented examples of CTG data in Supplementary Figure S1.

### Outcome labeling

The collected CTG data were segmented into 5-minute intervals for labeling. We labeled the data whether normal or abnormal. We defined late decelerations, variable decelerations, absent FHR variability, and sinusoidal patterns as abnormal CTG which requires obstetric intervention^13^.

To address the well-known issue of inter-observer disagreement in CTG interpretation^14,15^, we established a 2-stage reading system. For each hospital, two board-certified obstetricians from hospital labeled the CTG independently. In cases of disagreement between the labels provided by the two primary board-certified obstetricians, a senior obstetrician with over 15 years of experience made the final interpretation.

### Model development

We used the dataset from 11 hospitals for model development and internal testing. To prove the robustness of the deep learning model, we used the dataset from three hospitals for external testing. For model development and internal validation, we used 17,494 singleton deliveries and 1,246 multiple deliveries from the 11 hospitals, while for external validation, we used 3,155 singleton deliveries and 716 multiple deliveries. We randomly divided deliveries in an 8:1:1 ratio into training, validation, and internal test sets. No additional class imbalance correction was applied, as our development dataset included 436,695 person-minutes of CTG, with 15.9% labeled as abnormal—substantially higher than in previous studies^5–7^.

We used FHR, uterine contraction waveform data from CTG. We also extracted time-series features from CTG. From the extracted time-series features, we selected seven that, when incorporated, resulted in an AUC improvement of at least 0.001 on the validation dataset. We concated waveform and time-series features as input for the deep learning model. For model selection, we tested several deep learning algorithms, and selected SE-ResNet50 as the optimal model. SE-ResNet50 is a deep learning model that integrates the Squeeze-and-Excitation (SE)^16^ module into the ResNet50 architecture. ResNet50^17^ consists of 50 layers with residual blocks, where each block includes a convolutional layer, batch normalization^18^, and ReLU^19^ activation. The SE module in SE-ResNet50 helps the model enhance important features and suppress less relevant ones by learning channel-wise relationships, leading to improved performance. Detailed information about the model structure and model selection is provided in Supplementary Figure S2 and Supplementary Figure S3.

For metrics, we used the area under the receiver operating characteristic curve (AUC). In addition, we calculated the sensitivity, specificity, positive predictive value, and negative predictive value with a cut-off point from Youden’s J statistics in the development dataset. We utilized the DeLong test to assess the statistical significance of the AUC of our model.

We also evaluated model calibration to further assess model explainability. We generated calibration plots to examine how well the predicted probabilities align with the actual outcomes, offering additional insights into the model’s reliability and performance.

### Sensitivity analysis

To assess the robustness of the developed deep learning model, a subgroup analysis was conducted by categorizing patients into six clinically relevant groups. The subgroups were defined based on prematurity, emergent operations, and multiple pregnancies. The analysis was performed using internal test data and three external test datasets, enabling a comprehensive evaluation of the model’s performance stability within each subgroup.

### Statistical analysis

For baseline characteristics, both continuous and categorical variables are presented as mean values and absolute standardized differences. Continuous and categorical variables were compared accordingly. Some characteristics included missing values; in such cases, we calculated baseline characteristics using only complete cases. Python 3.8, Pytorch 1.11, Tsfresh 0.20, scikit-learn 1.3 was used for signal preprocessing and model development.

## Results

### Study cohorts

**Fig. 1:**
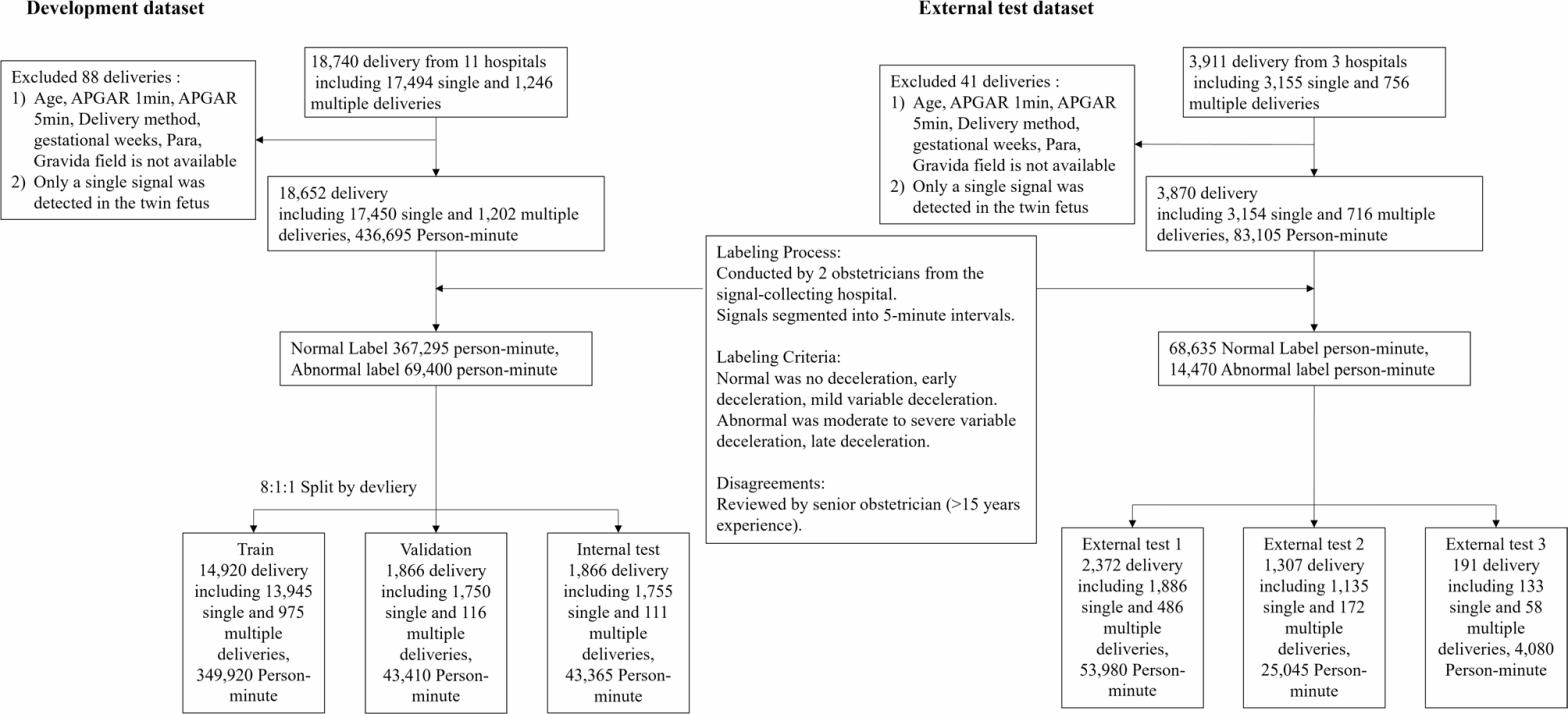
Study flowchart. The figure illustrates the process of data preparation and labeling for the development and external test datasets. The development dataset includes deliveries from 11 hospitals, with specific exclusion criteria and an 8:1:1 split into training, validation, and internal test sets. The external test dataset is derived from 3 hospitals and divided into three subsets after applying similar exclusion criteria. A two-stage labeling process was conducted by obstetricians using 5-minute signal intervals, categorizing labels as normal or abnormal based on specific criteria. Disagreements were resolved by senior obstetricians with over 15 years of experience.

The development dataset included 18,740 deliveries from 11 hospitals, while the external test dataset included 3,911 deliveries from 3 hospitals. After applying exclusion criteria, 88 deliveries were excluded from the development dataset and 41 deliveries were excluded from the external test dataset. As a result, a total of 22,522 deliveries and 519,800 person-minutes of CTG data were included in the study. After the labeling process, the development dataset contained a total of 436,695 person-minutes of CTG, of which 69,400 person-minutes (15.9%) were labeled as abnormal. The external test dataset contained a total of 83,105 person-minutes of CTG, with 14,470 person-minutes (17.4%) labeled as abnormal (Fig. 1).

We investigated baseline characteristics of patients (Table 1). In the developmental dataset, maternal age was similar between the abnormal and normal groups, with means of 32.9 and 33.1 years, respectively (ASD = 0.034). Among maternal factors, pre-eclampsia was more frequent in the abnormal group (11.4%) compared to the normal group (8.0%), with an ASD of 0.114, indicating a noticeable difference. Additionally, the cesarean section rate was lower in the abnormal group (54.5%) compared to the normal group (64.3%), with an ASD of 0.200, suggesting a moderate difference. For fetal characteristics, the incidence of fetal growth restriction was higher in the abnormal group (14.1%) compared to the normal group (9.2%), with an ASD of 0.152. Neonatal head circumference was slightly smaller in the abnormal group (32.8 cm) compared to the normal group (33.1 cm), with an ASD of 0.082. Additionally, the Apgar scores at 1 min (7.3 vs. 7.7) and 5 min (8.7 vs. 8.9) were lower in the abnormal group, with ASDs of 0.242 and 0.209, respectively, indicating significant differences. Among neonatal outcomes, intubation was more frequent in the abnormal group (10.5%) compared to the normal group (5.9%), with an ASD of 0.169. Similarly, low birth weight was more prevalent in the abnormal group (34.6%) compared to the normal group (31.4%), with an ASD of 0.067. All other characteristics showed no statistically significant differences.

**Table 1:** Baseline characteristics of the study cohort.

Characteristics	Development dataset			External Test dataset		
	Abnormal	Normal	ASD	Abnormal	Normal	ASD
	Mean	Mean		Mean	Mean	
Total	5487	14,367		1305	3281	
Mother						
Age (year)^†^	32.9	33.1	0.034	33.2	33.6	0.090
Height (cm)^†^	161.2	161.3	0.021	160.9	161.3	0.062
Weight (kg)^†^	69.7	70.3	0.049	65.1	67.0	0.157
Gravida^†^	1.9	2.0	0.118	2.2	2.2	0.068
Para^†^	0.5	0.5	0.097	0.9	1.0	0.054
Gestational hypertension	4.7%	4.2%	0.023	7.6%	5.8%	0.071
Gestational diabetes	8.3%	8.9%	0.023	11.6%	10.1%	0.051
Pre-eclampsia	11.4%	8.0%	0.114	11.6%	8.4%	0.108
Cesarean section	54.5%	64.3%	0.200	53.5%	67.4%	0.288
Gestational weeks^†^	36.6	36.6	0.025	36.4	36.4	0.027
Fetal						
Fetal growth restriction	14.1%	9.2%	0.152	10.0%	7.5%	0.089
Baby sex (male)	52.3%	52.7%	0.008	48.6%	52.4%	0.077
Weight (g)^†^	2712.3	2795.2	0.117	2653.9	2705.8	0.075
Height (cm)^†^	47.0	47.3	0.074	47.9	48.2	0.072
Head circumference (cm)^†^	32.8	33.1	0.082	32.6	33.1	0.184
Apgar score.1 min^†^	7.3	7.7	0.242	7.6	8.1	0.263
Apgar score.5 min^†^	8.7	8.9	0.209	8.8	9.1	0.215
NICU admission	46.6%	43.2%	0.068	42.6%	40.5%	0.043
Intubation	10.5%	5.9%	0.169	3.6%	1.5%	0.132
Jaundice	20.6%	20.3%	0.007	31.2%	32.9%	0.038
Prematurity	39.7%	39.7%	0.002	42.5%	42.6%	0.001
Low birth weight	34.6%	31.4%	0.067	39.2%	35.7%	0.072

^†^Means continous variable. The numbers of patients in each group before and after propensity score matching are presented, with percentages in parentheses. NICU, neonatal intensive care unit; ASD, absolute standardized difference;

### Performance of deep learning model

**Fig. 2:**
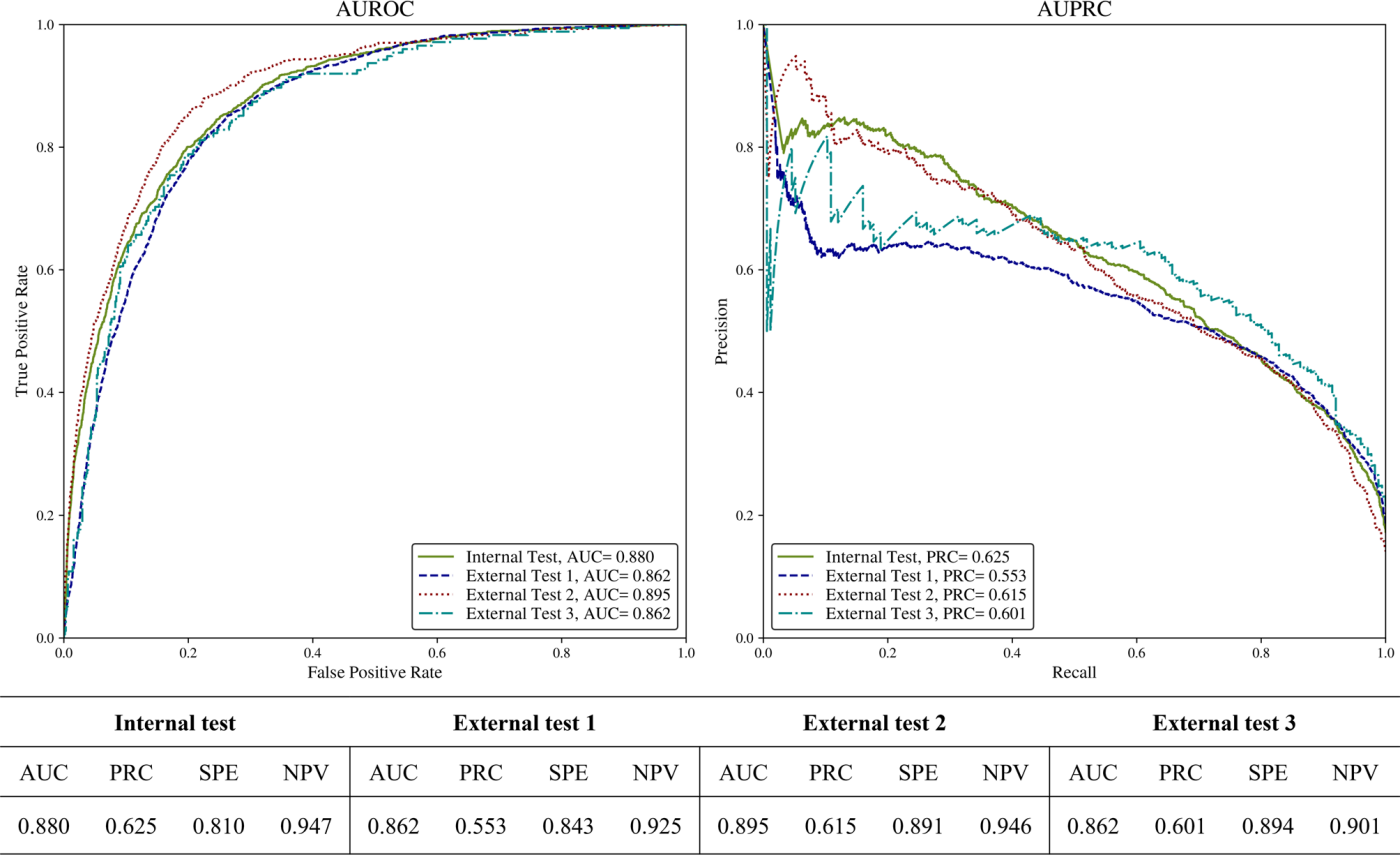
Model performance. The figure shows the performance metrics of the model evaluated on internal and external test datasets using AUC and PRC curves. The left plot represents the AUC (Area Under the Receiver Operating Characteristic curve), indicating the model’s discrimination ability across internal and external test sets. The right plot illustrates the PRC (Precision-Recall Curve), highlighting the model’s performance in detecting abnormal cases. The accompanying table summarizes key metrics, including AUC, PRC, specificity (SPE), and negative predictive value (NPV), for each dataset. Consistent performance across internal and external tests demonstrates the model’s generalizability.

The performance of the model was evaluated through both internal and external validation tests, with the results summarized in the AUC (Area Under the receiver operating characteristic Curve) and PRC (area under the Precision-Recall Curve). For the internal test, the model achieved an AUC of 0.880 and a PRC of 0.625, indicating strong overall performance in distinguishing between classes with high specificity (0.810) and negative predictive value (0.947). External test 1 yielded an AUC of 0.862 and a PRC of 0.553, while external test 2 showed an improved AUC of 0.895 and PRC of 0.615, demonstrating the model’s robustness across different datasets. External test 3 resulted in an AUC of 0.862 and a PRC of 0.601, further affirming the model’s consistent performance. The high NPV values across all tests (0.947 for internal, 0.901 to 0.946 for external) reflect the model’s strong ability to rule out false positives. These results are depicted in the AUC and PRC curves above (Fig. 2).

### Sensitivity analysis

Through the AUC results for the entire dataset and subgroup analyses, it was observed that the model exhibited relatively consistent performance across diverse patient characteristics. In the internal test dataset, the model achieved a generally high AUC value (above 0.880), and similar levels of AUC were maintained across most subgroups in the external test dataset. This suggests that the model maintains a certain level of predictive performance even in external environments and is applicable to a variety of patient populations (Table 2).

**Table 2:** Model performance in sensitivity analysis.

Subgroup	Internal	External 1	External 2	External 3
Preterm baby	0.879	0.868	0.893	0.844
Full term baby	0.880	0.859	0.892	0.892
Emergency delivery	0.882	0.878	0.902	0.859
Elective delivery	0.877	0.839	0.867	0.870
Singleton	0.881	0.853	0.907	0.875
Multiple	0.869	0.861	0.860	0.843

The table presents the sensitivity analysis results, showing the model’s AUC across various subgroups, including preterm and full-term babies, emergency and elective deliveries, and singleton and multiple deliveries. The consistent AUC values across internal and external datasets demonstrate the model’s robustness and generalizability across different clinical scenarios. Detailed information about Sensitivity analysis metrics are provided in Supplementary Table S1 and Supplementary Table S2.

Particularly, subgroups such as preterm babies, emergency deliveries, and singleton pregnancies showed high AUC values, indicating stable predictive performance in these groups. On the other hand, subgroups such as multiple pregnancies and elective deliveries showed relatively lower AUC values in some external tests, suggesting that the predictive performance may decrease for these groups. However, overall, all subgroups maintained AUC values above 0.84, demonstrating that the model provides reliable predictive performance across various clinical conditions. As a result of the DeLong test, the P-value was < 0.01 in all subgroups.

### Model calibration

The calibration metrics of our model indicate the agreement between predicted probabilities and observed outcome rates for both internal and external test datasets. In the calibration plot, the diagonal dashed line represents a perfect calibration line, which would occur if predicted probabilities matched the observed outcome probabilities perfectly.

**Fig. 3:**
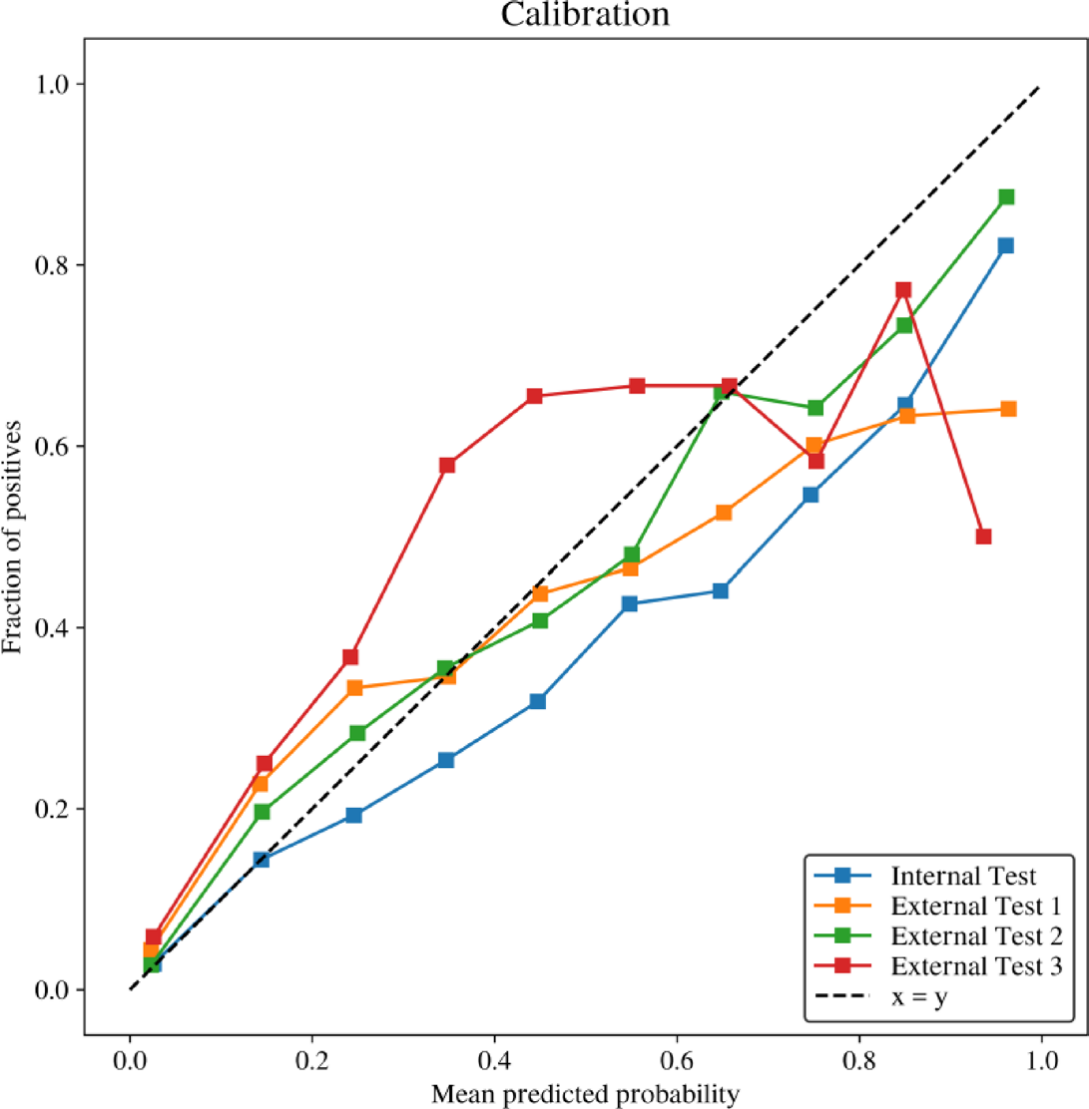
Model calibration. The calibration plot shows the agreement between predicted probabilities and observed outcomes for the internal test and three external test datasets. The x-axis represents the mean predicted probability, while the y-axis indicates the fraction of positive cases. The dashed diagonal line (x = y) represents perfect calibration, where predictions match the observed outcomes exactly. The model demonstrates varying levels of calibration across datasets, with predictions generally aligning closer to the diagonal in some datasets than others. This highlights the model’s calibration performance and its ability to provide reliable probability estimates.

The calibration curves for internal testing and external tests 1, 2, and 3 show some overestimation or underestimation in certain segments, particularly with external test 3 in the high-probability region, where predicted probabilities tend to be overestimated compared to actual outcome rates. Nevertheless, the overall trend of the calibration curves is close to the x = y line, indicating adequate calibration of the model (Fig. 3).

## Discussion

In our study, data from 22,522 deliveries and 519,800 person-minutes of CTG collected across 14 hospitals were utilized to develop a deep learning model, which was validated through internal and external testing. The model achieved an AUC of 0.880 and a PRC of 0.625 in internal testing, while in external testing across three hospitals, it demonstrated AUCs of 0.862, 0.895, and 0.862 and PRCs of 0.553, 0.615, and 0.601, respectively, demonstrating its generalizability. Given the limitations of previous CTG automated interpretation systems, which were constrained by limited datasets and low accuracy, our study holds significant value in improving model generalization through the use of large-scale, multi-institutional data and robust study design.

Additionally, in all sub-analyses, the model demonstrated stable performance, achieving an AUC of 0.869 in internal validation and exceeding an AUC of 0.839 in external test. Notably, in subgroups with high clinical relevance, such as preterm infants and emergency deliveries, the model achieved AUCs of 0.879 and 0.882, respectively, underscoring its clinical utility. Furthermore, calibration plot shows that the model’s abnormal predictions were aligned with the distribution of actual abnormal cases.

Our model combines deep learning with traditional signal processing features, and there are a couple of reasons for this approach. First, since our study is based on convolutional networks, the model excels at capturing the local characteristics of individual signals but faces challenges in understanding the overall characteristics of the entire signal at once. Second, with SeResNet50 having 28 million parameters, the 100,000 data points used in this study may have been insufficient to fully optimize the deep learning model. Therefore, incorporating traditional signal processing features may have helped optimize the model by providing additional relevant information that the deep learning model could not learn from the raw data alone.

Our study has two major strengths compared to prior CTG automatic interpretation studies. First, it significantly expanded the number of participating patients and hospitals. While prior studies use fewer than 5,000 patients, our study utilized a large-scale, multi-institutional dataset comprising 22,522 delivery records collected from 14 hospitals. This extensive dataset facilitated the model in demonstrating excellent model performance. Secondly, we conducted an extensive labeling process to create a dataset aligned with the conventional clinical use of CTG. While previous studies primarily relied on extractable test variables, such as laboratory results, our approach involved direct expert labeling by multiple obstetricians. To address potential disagreements among labelers, we implemented a two-stage labeling process to enhance precision. Initially, two obstetricians from each hospital independently analyzed the data. In cases of disagreement, a senior obstetrician with over 15 years of experience resolved the discrepancy, ensuring high accuracy and reliability in the labeled data. The comparison between our study and previous studies is summarized in Table 3.

**Table 3:** The comparison between our study and previous studies.

Study ID	Pre-processing method	Type of features extracted	Part of CTG used	Clinicians as author(s)	Hold-out-validation	Cross validation method	ML classifier(s) used	Model interpretability	Performance measure(s)	Oversampling
Our study	Excluded records with missing data (e.g., maternal age, Apgar scores) or incomplete twin signals (> 1-min discontinuity)	From 0.5 Hz time-point signals, 7 statistical features (max, min, median, mean, number of peaks, variance, standard deviation) were extracted for the final model	FHR and UC	Both	Yes	None	1D-SEResNet50	No	SEResNet50:	No
									Specificity = 81%	
									NPV = 94.7%	
									AUPRC = 62.5%	
									AUROC = 88%	
^20^	Linear interpolation	FHR FIGO features and UC	FHR and UC	Non-clinician	Yes	k-fold	LG	Yes	LG:	No
									AUROC = 74%	
^21^	Did not specify	Morphological	FHR and UC	Non-clinician	No	k-fold	MLP, bagging, RF ands SVM	Partially	RF	Yes
									Sensitivity = 96.4%	
									Specificity = 98.4% Accuracy = 96.7%	
									Precision = 96.8%	
^22^	Remove spikes, interpolate, and segment into 20 min	Wavelet packet decomposition image	FHR	Non-clinician	No	k-fold	2DCNN	No	CNN:	No
									Accuracy = 95.24% Sensitivity = 90.4% Specificity = 100%	
^23^	Smoothing	Morphological and statistical	FHR and UC	Non-clinician	No	k-fold	NN, RF, clustering and SVM	Partially	Ensemble combination- NN, RF, k-means and SVM: Accuracy = 92.30%	No
^24^	Processing outliers and removing spike using moving average	Image	FHR and UC	Non-clinician	Yes	Did not specify	1D-CNN and bidirectional Gate	No	Accuracy = 95.15%	No
									Sensitivity = 96.20% Specificity = 94.09%, Precision = 94.21%	
							Recurrent Unit (BiGRU)		F measure = 95.20%	
									AUROC = 99.29%	
^25^	Outlier detection and linear interpolation	Linear and nonlinear, extract feature using CNN & LSTM	FHR	Non-clinician	No	k-fold	SVM and CNN-BiLSTM	Partially	SVM:	No
									Sensitivity = 56.97% Specificity = 73.35%	
									QI = 63.91%	
^26^	Did not specify	Image based and text	FHR	Non-clinician	Yes	Stratified k-fold	CNN	No	MMIF-1 (ViT-B/16): Accuracy = 96.3%	No
									F measure = 96.3%	
									AUROC = 96.2%	
^27^	Did not specify	Image	FHR	Non-clinician	Yes	k-fold	KNN, NB, SVM, DT, RF, ADABOOST, XGBOOST	No	XGBOOST:	No
									Accuracy = 96.3%	
									Precision = 95.4%	
									Recall = 97.3%	
									F measure = 96.4%	
									AUROC = 95.9%	
^28^	Lagrange interpolation	Image	FHR	Non-clinician	No	Did not specify	Double Trend Accumulation Former CNN	No	Accuracy = 90.6%	No
^29^	Lagrange interpolation	Curve classification	FHR	Non-clinician	Yes	k-fold	Trend-Guided Long CNN	No	Accuracy = 89.80%	No

While existing models using objective outcomes such as pH provide meaningful clinical value, their focus often diverges from real-time CTG interpretation tasks that are integral to current intrapartum workflows^15,16^. Our model instead targets the recognition of standardized patterns—such as late, variable, and prolonged decelerations—which are routinely assessed by obstetricians. These two different approaches offer potential as an automated alarm system embedded within ongoing fetal monitoring. In the future, alarm systems that incorporate a wider range of clinically relevant features will be essential for real-world implementation in delivery rooms.

Our study has several limitations. First, as it is based on a retrospective design, the model’s effectiveness in real-time clinical settings has not been validated. Prospective studies are necessary to confirm its practical utility in improving fetal and maternal outcomes during labor. Also, the study collected CTG data from image (PNG) files, and due to image resolution limitations, only 0.5 Hz data was extracted instead of the original 4 Hz signal This limitation may have restricted the deep learning model’s ability to learn intricate features of complex CTG signals. If higher-resolution original waveform data were available, the model performance could be improved. Future study should aim to improve access to original waveform data or explore new data processing methods to minimize resolution loss. Another limitation of this study is that relatively short CTG segments, ranging from 5 to 75 min, were examined as sequences of PNG files. The necessity of obstetricians for labeling, particularly to address inter-labeler discrepancies, limited the number of labeled signals. In order to increase the patient count, it was necessary to restrict the signal duration per patient. Future studies should aim to involve more labelers, allowing for an increase in both the signal duration per patient and the overall dataset size. Also, Inter-labeler variability could raise concerns about labeling reliability. To mitigate this, we used a 2-stage labeling process with two clinicians, followed by a final review from a clinician with over 15 years of experience. Nevertheless, the subjectivity of labeling remains a limitation.

Future studies should focus on validating the effectiveness of our model through prospective clinical trials and expanding the dataset to include diverse patient groups, such as those with multiple pregnancies, where performance was relatively lower. Also, rather than merely analyzing CTG in 5-minute intervals, interpreting continuous signals as time-series data could lead to a more clinically relevant model. Incorporating such time-series analysis techniques may enhance the model’s predictive power. Through these improvements, the model has the potential to establish a new standard for CTG interpretation during labor, serving as a critical tool for early detection of fetal hypoxemia and improving outcomes for both mothers and fetuses.

## Conclusion

Our study demonstrates that a deep learning-based model can achieve high diagnostic performance in automated CTG interpretation, leveraging a large-scale, nationwide dataset with expert-annotated labels. Future prospective studies are needed to validate its clinical applicability and potential to improve fetal prognosis.

## Electronic supplementary material

Below is the link to the electronic supplementary material.

Supplementary Material 1

## Data Availability

The dataset used in this study is a public dataset with limited access that can be used after approval by the National Information Society Agency (NIA), and details can be found on the AI-Hub website (https://www.aihub.or.kr/aihubdata/data/view.do? currMenu=115&topMenu=100&aihubDataSe=data&dataSetSn=71366).

## Abbreviations

CTG: CardioTocoGraphy
FHR: Fetal heart rate
UC: Uterin contraction
EMR: Electronic medical records
AUC: Area under the receiver operating characteristic curve
PRC: Area under the precision-recall curve
