# Supplementary material for: Automated interpretation of cardiotocography using deep learning in a nationwide multicenter study
Source: Sci Rep. 2025 Jun 4;15:19617. doi: 10.1038/s41598-025-02849-4 (PMC12137656; doi:10.1038/s41598-025-02849-4)
Supplement: Supplementary file 1 — Supplementary Material 1 [file 41598_2025_2849_MOESM1_ESM.docx]

**Supplementary Materials**

**Supplementary Note**

**Supplementary Note. Participating hospitals**

In 2022, South Korea, in collaboration with the National Information Society Agency (NIA), launched a nation-wide, multi-center project to build a dataset for cardiotocography, aiming to develop AI models for detecting abnormal fetal conditions during pregnancy.

NIA dataset 14 hospitals in South Korea.

Development dataset included 11 hospitals :

Ajou University Medical Center (4,903 patients, IRB: AJIRB-MED-MDB-21-715),

Seoul St. Mary’s Hospital (1,127 patients, IRB: KC22RNDI0316),

Kyungpook National University Chilgok Hospital (2,364 patients, IRB: KNUCH 2022-04-027),

Kyungpook National University Hospital (457 patients, IRB: KNUH 2022-04-023)

Kyung Hee University Hospital (2,366 patients, IRB: KNUH 2022-04-023),

Korea University Medical Center (681 patients, IRB: 2022AS0143),

Dankook University Hospital (2,074 patients, IRB: DKUH 2202-04-008),

Inje University Paik Hospitals (Busan, Haeundae, Ilsan - total 3,663 patients, IRB: BPIRB 2022-05-003, HPIRB 2022-05-046-001, ISPAIK 2022-05-016),

Seoul National University Hospital (1,105 patients, IRB: H2208-006-1346),

External test dataset included 3 hospitals :

Seoul National University Bundang Hospital (2,382 patients, IRB: B-2204-752-103),

Chonnam National University Hospital (1,338 patients, IRB: CNUH-2022-179),

Chung-Ang University Hospital (191 patients, IRB: 2206-012-19423).

**Supplementary Figures**

**Figure S1. Examples of Cardiotocography**


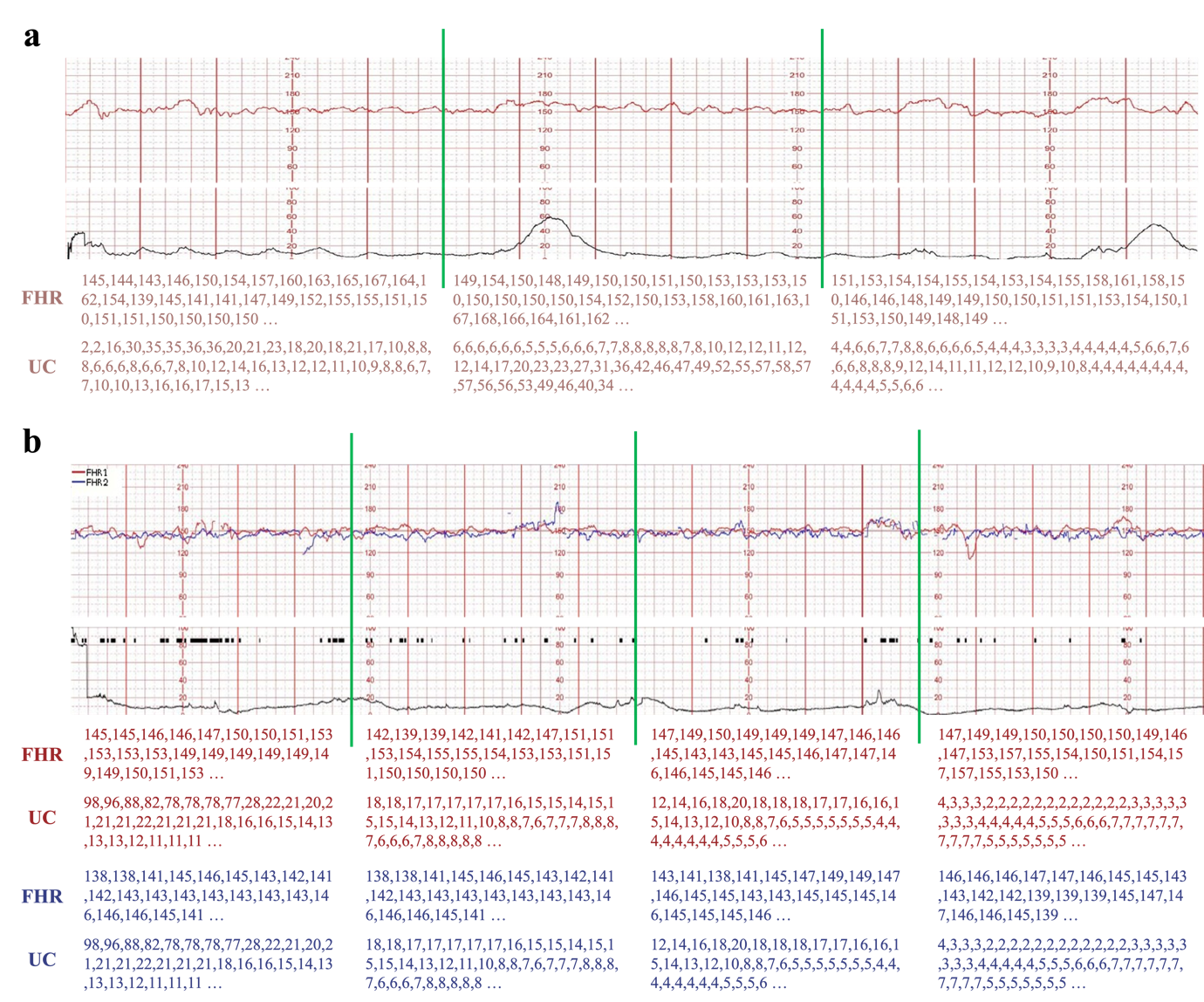


Examples of CTG images for singleton (a) and multiple pregnancies (b). Each segment was extracted at 0.5 Hz using the Hough transform algorithm and contains FHR and UC signals with 150 time points.

**Figure S2. Model selection**


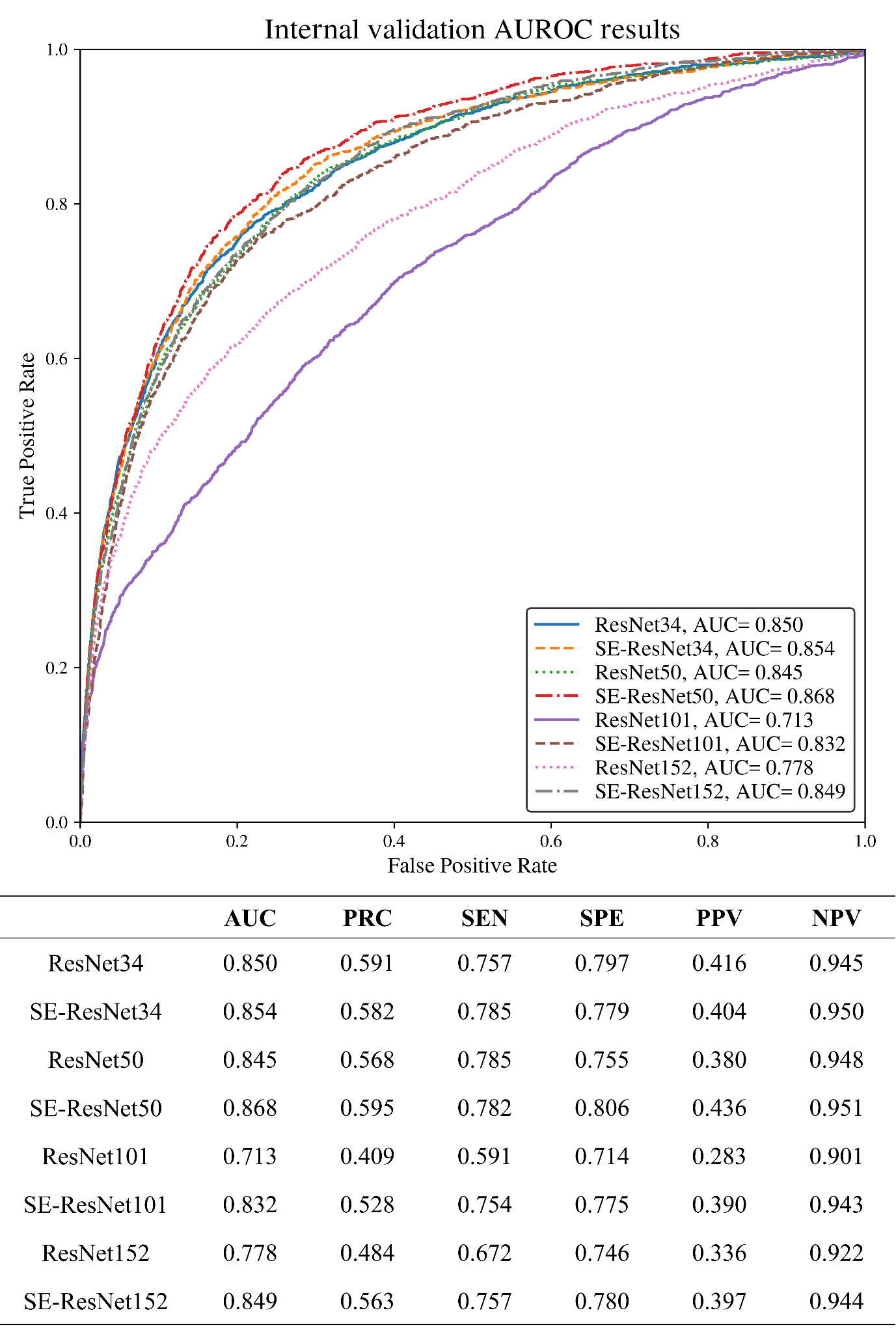


In the preliminary experiments, classification performance was found to be insufficient when relying solely on raw waveform signals. To address this, the python tsfresh package was utilized to extract temporal features from the raw waveforms, which substantially improved the model's classification capabilities(1, 2). The features extracted included maximum, minimum, median, mean, number of peaks, variance, and standard deviation. Additionally, Z-normalization was applied to the raw waveform signals to scale numerical values appropriately(3). This preprocessing prevented model bias associated with weight adjustments. After these preprocessing steps, there was a notable enhancement in the model's key performance indicators, such as AUC and accuracy.

Regarding the classification models, both XGBoost(4) and ResNet(5) models were evaluated. Due to the requirements for multichannel signal processing, XGBoost was inappropriate and consequently excluded from consideration. During internal validation, comparisons among various ResNet models indicated that the SE-ResNet50 model achieved the highest scores in AUC and PRC, demonstrating its superior efficacy in predicting the positive (abnormal) class. It also exhibited high specificity and NPV, ensuring its reliability in predicting normal cases. As a result, SE-ResNet50 was selected as the most suitable model.

We compared the internal validation results of ResNet models using performance metrics, including AUC, PRC, sensitivity, specificity, PPV, and NPV. Among the models evaluated, the SE-ResNet50 model achieved the highest AUC and PRC, indicating superior performance in predicting the positive (abnormal) class. Additionally, it demonstrated high specificity and NPV, confirming its reliability in predicting normal cases. According to comparison, SE-ResNet50 was selected as the most appropriate model.

**Figure S3. Model structure**


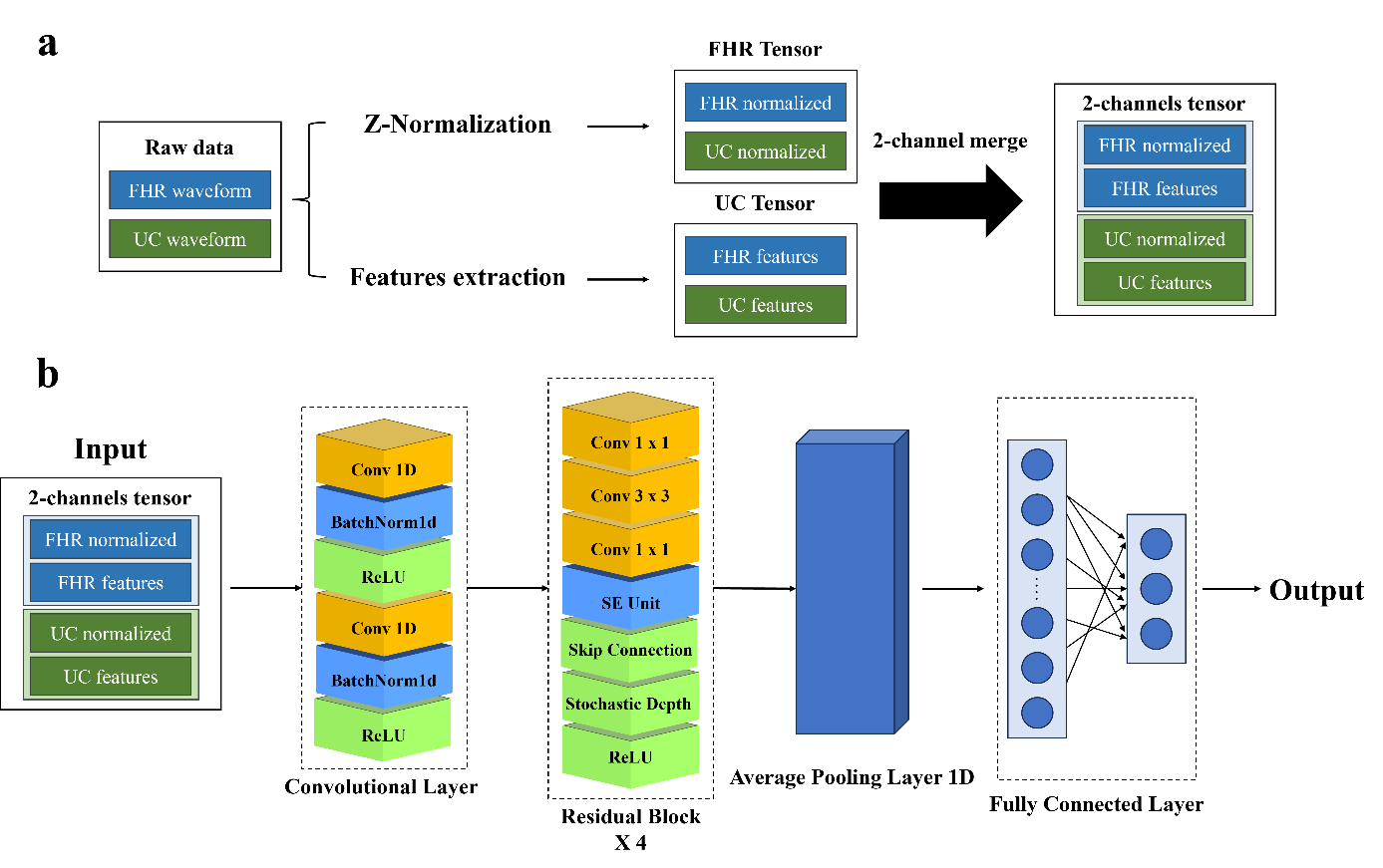


In our study, we propose a SE-ResNet50 model modified to fit the 1D data to process FHR and UC signals. The architecture of the model is presented above.

We constructed 1D-SE-ResNet50, which comprises a Convolutional layer, Residual block, Average pooling layer 1D, and a Fully connected layer. The model receives the FHR, UC signals at 150 time points and 7 extruded features through 2-channel. Z-normalization was applied to the FHR and UC signals.

The Convolutional layer performs 1D convolution operations to extract features along the time axis, using 7x7 and 3x3 filters to detect features of different scales and incorporating the ReLU(6) activation function to introduce non-linearity, allowing the model to learn complex patterns. The Residual blocks are structured similarly to the ResNet50 architecture, consisting of 3, 4, 6, and 3 bottleneck blocks in each stage. Each bottleneck block includes three convolutional units for channel reduction, feature learning, and channel expansion, along with an SE Unit(7), skip connections, stochastic depth, and ReLU activation, which together stabilize training, deepen the network, and enhance generalization. Especially, the SE Unit recalibrates the feature maps to emphasize the importance of each channel. The Average pooling layer 1D reduces the size of the feature maps by averaging the information (adaptive average pooling), refining the features into the most important representations, and reducing the computational cost(8), while the Fully connected layer connects the final output of the model to determine the classification of normal and abnormal cases, making the final prediction.

We used the Adam optimizer with a learning rate of 0.0001. We adopted BCEWithLogitsLoss to handle binary classification by applying a sigmoid function internally. The model was trained for up to 100 epochs, and training was early-stopped if the validation loss did not improve by at least 0.0001 compared to the best recorded validation loss for 10 consecutive epochs.

**Supplementary Table**

**Table S1. Data employed in the Sensitivity analysis**

Below table shows Number of fetuses for each subgroup (per birth)

|  | Internal Test | External 1 | External 2 | External 3 |
| --- | --- | --- | --- | --- |
| Preterm | 762 | 984 | 868 | 143 |
| Full term | 1215 | 1874 | 611 | 106 |
| Total | 1977 | 2858 | 1479 | 249 |
|  |  |  |  |  |
| Emergency | 1187 | 1548 | 1195 | 167 |
| Elective | 790 | 1310 | 284 | 82 |
| Total | 1977 | 2858 | 1479 | 249 |
|  |  |  |  |  |
| Singleton | 1755 | 1886 | 1135 | 133 |
| Multiple | 222 | 972 | 344 | 116 |
| Total | 1977 | 2858 | 1479 | 249 |

Multiple deliveries have two births each.

Below table shows Number of normal/abnormal labels (per 5 minutes) :

| **Abnormality** | **Train** | **Validation** |
| --- | --- | --- |
| Normal | 59,002 (84.3) | 7,288 (83.9) |
| Abnormal | 10,982 (15.7) | 1,394 (16.1) |

|  |  | | | |
| --- | --- | --- | --- | --- |
|  | **Internal Test** | **External Test 1** | **External Test 2** | **External Test 3** |
| Development |  |  |  |  |
| Normal | 7,169 (82.7) | 8,782 (81.3) | 4,304 (85.9) | 641 (78.6) |
| Abnormal | 1,504 (17.3) | 2,014 (18.7) | 705 (14.1) | 175 (21.4) |
| Preterm baby |  |  |  |  |
| Normal | 2,713 (81.9) | 2,981 (81.6) | 2,518 (83.6) | 349 (78.8) |
| Abnormal | 601 (18.1) | 671 (18.4) | 493 (16.4) | 94 (21.2) |
| Full term baby |  |  |  |  |
| Normal | 4,456 (83.1) | 5,801 (81.2) | 1,786 (89.4) | 292 (78.3) |
| Abnormal | 903 (16.9) | 1343 (18.8) | 212 (10.6) | 81 (21.7) |
| Emergency delivery |  |  |  |  |
| Normal | 4,253 (83.8) | 4,711 (85.1) | 3,511 (86.9) | 407 (78.7) |
| Abnormal | 821 (16.2) | 824 (14.9) | 529 (13.1) | 110 (21.3) |
| Elective delivery |  |  |  |  |
| Normal | 2,916 (81) | 4,071 (77.4) | 793 (81.8) | 234 (78.3) |
| Abnormal | 683 (19) | 1,190 (22.6) | 176 (18.2) | 65 (21.7) |
| Singleton |  |  |  |  |
| Normal | 6421 (82.4) | 5,677 (77.8) | 3,211 (85.7) | 374 (77.6) |
| Abnormal | 1,372 (17.6) | 1,617 (22.2) | 534 (14.3) | 108 (22.4) |
| Multiple |  |  |  |  |
| Normal | 748 (85.0) | 3,105 (88.7) | 1,093 (86.5) | 267 (79.9) |
| Abnormal | 132 (15.0) | 397 (11.3) | 171 (13.5) | 67 (20.1) |

Data are presented as n (%).

**Table S2. Sensitivity analysis metrics by subgroup**

Below table shows Model performance for each subgroup :

|  | **Preterm baby** | | | | |  | **Full term baby** | | | | |
| --- | --- | --- | --- | --- | --- | --- | --- | --- | --- | --- | --- |
| **Dataset** | **AUC** | **SEN** | **SPE** | **PPV** | **NPV** |  | **AUC** | **SEN** | **SPE** | **PPV** | **NPV** |
| **Internal Test** | 0.879 | 0.782 | 0.808 | 0.475 | 0.944 |  | 0.880 | 0.788 | 0.812 | 0.459 | 0.950 |
| **External Test 1** | 0.868 | 0.683 | 0.874 | 0.549 | 0.924 |  | 0.859 | 0.713 | 0.827 | 0.489 | 0.926 |
| **External Test 2** | 0.893 | 0.704 | 0.877 | 0.529 | 0.938 |  | 0.892 | 0.660 | 0.911 | 0.468 | 0.958 |
| **External Test 3** | 0.844 | 0.532 | 0.917 | 0.633 | 0.879 |  | 0.892 | 0.765 | 0.866 | 0.614 | 0.930 |

|  | **Emergency delivery** | | | | |  | **Elective delivery** | | | | |
| --- | --- | --- | --- | --- | --- | --- | --- | --- | --- | --- | --- |
| **Dataset** | **AUC** | **SEN** | **SPE** | **PPV** | **NPV** |  | **AUC** | **SEN** | **SPE** | **PPV** | **NPV** |
| **Internal Test** | 0.882 | 0.775 | 0.825 | 0.461 | 0.950 |  | 0.877 | 0.799 | 0.789 | 0.471 | 0.944 |
| **External Test 1** | 0.878 | 0.655 | 0.888 | 0.506 | 0.936 |  | 0.839 | 0.735 | 0.791 | 0.507 | 0.911 |
| **External Test 2** | 0.902 | 0.686 | 0.901 | 0.511 | 0.950 |  | 0.867 | 0.705 | 0.849 | 0.508 | 0.928 |
| **External Test 3** | 0.859 | 0.627 | 0.907 | 0.645 | 0.900 |  | 0.870 | 0.662 | 0.872 | 0.589 | 0.903 |

|  | **Singleton** | | | | |  | **Multiple** | | | | |
| --- | --- | --- | --- | --- | --- | --- | --- | --- | --- | --- | --- |
| **Dataset** | **AUC** | **SEN** | **SPE** | **PPV** | **NPV** |  | **AUC** | **SEN** | **SPE** | **PPV** | **NPV** |
| **Internal Test** | 0.881 | 0.789 | 0.811 | 0.472 | 0.947 |  | 0.869 | 0.758 | 0.802 | 0.403 | 0.949 |
| **External Test 1** | 0.853 | 0.727 | 0.816 | 0.530 | 0.913 |  | 0.861 | 0.605 | 0.892 | 0.417 | 0.946 |
| **External Test 2** | 0.907 | 0.691 | 0.907 | 0.552 | 0.946 |  | 0.860 | 0.690 | 0.846 | 0.413 | 0.946 |
| **External Test 3** | 0.875 | 0.694 | 0.874 | 0.615 | 0.908 |  | 0.843 | 0.552 | 0.921 | 0.638 | 0.891 |

**REFERENCES**

1. Christ M, Braun N, Neuffer J, Kempa-Liehr AW. Time series feature extraction on basis of scalable hypothesis tests (tsfresh–a python package). Neurocomputing. 2018;307:72-7.

2. Christ M, Kempa-Liehr AW, Feindt M. Distributed and parallel time series feature extraction for industrial big data applications. arXiv preprint arXiv:161007717. 2016.

3. Passalis N, Tefas A, Kanniainen J, Gabbouj M, Iosifidis A. Deep adaptive input normalization for time series forecasting. IEEE transactions on neural networks and learning systems. 2019;31(9):3760-5.

4. Chen T, Guestrin C, editors. Xgboost: A scalable tree boosting system. Proceedings of the 22nd acm sigkdd international conference on knowledge discovery and data mining; 2016.

5. He K, Zhang X, Ren S, Sun J, editors. Deep residual learning for image recognition. Proceedings of the IEEE conference on computer vision and pattern recognition; 2016.

6. Nair V, Hinton GE, editors. Rectified linear units improve restricted boltzmann machines. Proceedings of the 27th international conference on machine learning (ICML-10); 2010.

7. Hu J, Shen L, Sun G, editors. Squeeze-and-excitation networks. Proceedings of the IEEE conference on computer vision and pattern recognition; 2018.

8. Krizhevsky A, Sutskever I, Hinton GE. ImageNet classification with deep convolutional neural networks. Communications of the ACM. 2017;60(6):84-90.
